# Epidemiology and treatment of distal radius fractures in Reykjavik, Iceland, in 2004: Comparison with an Icelandic study from 1985

**DOI:** 10.3109/17453674.2011.606074

**Published:** 2011-09-02

**Authors:** Kristbjörg Sigurdardottir, Sigurdur Halldorsson, Johann Robertsson

**Affiliations:** ^1^Department of Orthopedics, Falun Hospital, Falun, Sweden; ^2^The Health Center of Thingeyjarsyslur, Kopasker; ^3^Department of Orthopedics, Landspitali University Hospital, Reykjavik, Iceland; Correspondence: kristbjorg.sigurdardottir@ltdalarna.se

## Abstract

**Background and purpose:**

Recent literature suggests that the incidence and treatment modalities of distal radius fractures have been changing over the past 2 decades in the developed world. We examined the epidemiology of adult distal radius fractures in Iceland in 2004 and compared it with an Icelandic study from 1985 and other studies.

**Methods:**

A retrospective study of the epidemiology, classification, and treatment of distal radius fractures in Reykjavik residents aged 16 and older in 2004 was conducted by analysis of medical records and re-evaluation of all radiographic examinations.

**Results:**

The 228 fractures included in our material yielded an overall annual incidence of 17/10^4^ in men and 37/10^4^ in women. Age-specific incidence rose steadily with age in both sexes. One third of the fractures were intraarticular, and working-age men accounted for a large proportion of them. 95% of fractures were treated nonoperatively.

**Interpretation:**

The annual incidence of distal radius fractures was similar in 1985 and 2004. However, age-specific incidence in younger postmenopausal women decreased sharply. This trend has also been observed in recent Scandinavian studies. Most fractures were treated nonoperatively in Iceland in 2004.

Robertsson et al. (1990) examined the epidemiology of distal radius fractures among adults in the Reykjavik area in 1985. According to their study, and also other Scandinavian studies performed between 1979 and 1982 (Falch 1983, Solgaard and Peterson 1985, Schmalholz 1988), the incidence increased steadily with age, especially in postmenopausal women aged 50–70 years, but leveled off in older age groups. Some British and North American studies have shown similar results (Owen et al. 1982, O'Neill et al. 2001, Thompson et al. 2004). However, the recent literature from Scandinavia suggests a change in pattern over the past two decades, whereby the previous steep rise in incidence in women over 50 now occurs after 70 years of age (Brogren et al. 2007, Lofthus et al. 2008).

We examined the epidemiology of distal radius fractures in Reykjavik in 2004, in order to compare the results with those from the earlier Icelandic study from 1985 (Robertsson et al. 1990) and with those from international research.

## Patients and methods

The emergency and orthopedic departments of the largest hospital in Reykjavik—Landspitalinn (the National Hospital of Iceland)—diagnose and treat virtually all fractures sustained by residents of Reykjavik municipality (with 112,490 inhabitants in 2004). Even Reykjavik residents initially treated elsewhere in Iceland or abroad are almost always referred to the Landspitalinn orthopedic department for follow-up, and are therefore included in the hospital's diagnostic registry. In such cases, date and place of the accident are always documented.

Using ICD-10 codes S52.5 and S52.6, we searched the hospital's computerized medical record system (SAGA) for all patients living in Reykjavik municipality who had been diagnosed with a distal radius fracture from January 1 to December 31, 2004. Patients diagnosed at Landspitalinn, but living outside Reykjavik, were excluded. We used population figures from December 1, 2004 obtained from the Statistics Iceland website (Hagstofan).

The first author (KS) examined all patient medical records and gathered data on age, sex, time of accident, type of accident, and treatment. The first author also re-evaluated all radiological studies of the fractures and classified them according to two systems: the classic method (Colles', Barton's etc.) and the modified AO classification system. The third author was consulted in cases that were unclear.

The study was designed to allow comparison with the only earlier Icelandic epidemiological study on distal radius fractures from 1985 (Robertsson et al. 1990). We therefore used the same inclusion criteria, i.e. radius fracture within 3 cm of the radiocarpal joint in patients aged 16 and older. Our study population, residents of Reykjavik municipality aged 16 and older (n = 88,075), was similar to the population studied in 1985—inhabitants of the few municipalities that comprise the Reykjavik capital area, aged 16 and older (n = 100,154). We therefore considered that the results from these two studies would be comparable, which would allow us to draw valid conclusions with regard to change in incidence and treatment between 1985 and 2004. Unfortunately, we were unable to make an exact comparison of age-specific incidence and seasonal variation in the two studies because the exact data from the study by Robertson et al. had been lost. We could, however, approximate the data from figures in the original article and we do not consider this to be a serious drawback since we were able to make good estimates from the figures (Robertsson et al. 1990).

### Statistics

Incidence rates were age-standardized to allow comparison with other studies. The one-way chi-square test was used to compare incidence rates for age groups and fracture types.

The study was approved by the Data Protection Authority (Persónuvernd) and the National Bioethics Committee (Vísindasiðanefnd) of Iceland in December, 2006.

## Results

### Age and sex distribution

**Figure 1.**
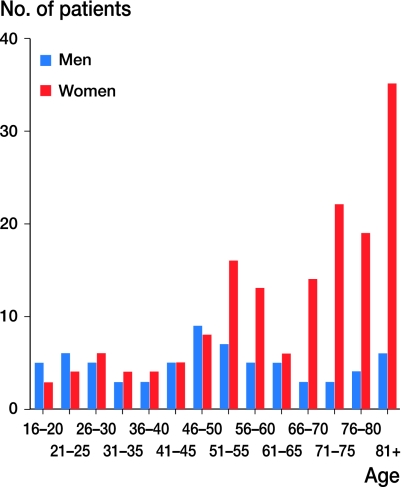
Age distribution of patients with distal radius fractures in Reykjavik in 2004.

The primary data search yielded 261 fractures. Re-examination of all radiographs showed that 33 patients did not have a distal radius fracture according to the inclusion criteria. The remaining 228 fractures occurred in 159 women (70%) and 69 men (Figure 1).

### Incidence

**Figure 2.**
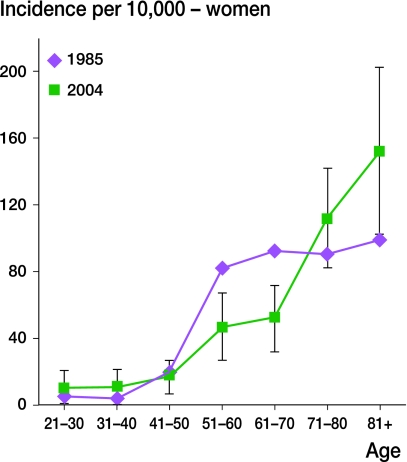
Age-specific incidence of distal radius fractures in women aged over 20 in 2004 (with 95% confidence intervals) compared with the 1985 study (Robertsson et al. 1990). (Here, age over 20 is used for comparison with the figure of Robertsson et al.). **^a^** (See Figure 4)

**Figure 3.**
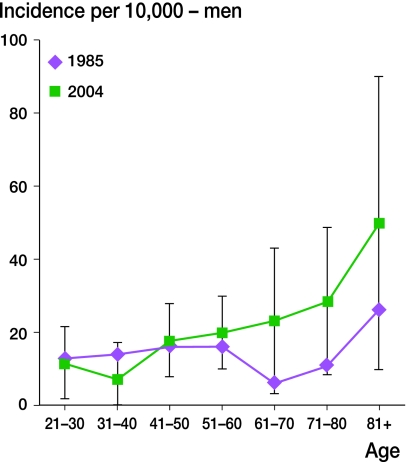
Age-specific incidence of distal radius fractures in men over 20 in 2004 (with 95% confidence intervals) compared with the 1985 study (Robertsson et al. 1990). (Here, age over 20 is used for comparison with the figure of Robertsson et al.). **^a^** (See Figure 4)

The total annual incidence was 27 (95% CI: 23–31) per 10^4^ persons, 37 (95% CI: 31–43) for women and 17 (95% CI: 13–21) for men. The mean age was 64 (SD 19) years for women and 50 (SD 20) years for men (Figures 2 and 3).

### Time and type of accident

**Figure 4.**
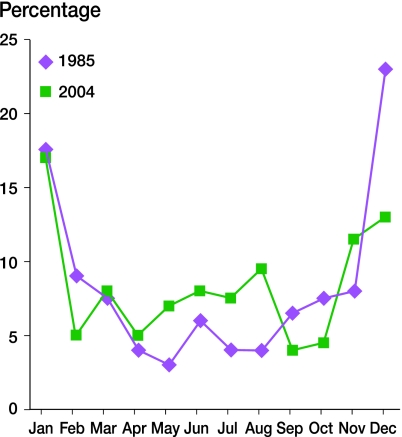
Seasonal variation of distal radius fractures in Reykjavik in 1985 and 2004. The figures from 1985 are not exact but approximated from figures from the original article (Robertsson et al. 1990).

54% of the fractures resulted from falling on level ground (30% indoors and 24% outdoors without icy conditions), 19% resulted from higher-level falls or falls during sports activities, and 18% resulted from falling due to icy conditions. 12% of the fractures occurred during working hours, 16% were sports-related, and 50% happened during other leisure activities. The highest incidence of fractures was during the winter months (November to February) (Figure 4).

### Classification

77% were Colles' fractures, 6% were Chauffeur's fractures, 1.5% were dorsal Barton's fractures, 1.5% were die-punch fractures, and 1% were Smith's fractures. According to the modified AO classification system, two-thirds of the fractures were extraarticular, i.e. type A fractures (A2: 37%; A3: 29%), while 9% were type B (B1: 7%; B2: 2%) and 25% were type C (C1: 5%; C2: 15%; C3: 5%). The type A fractures had mainly resulted from falling on level ground with or without icy conditions (87%) while a third of the type C fractures had resulted from higher-level falls or falls during sports activities (Table).

**Table:** Number of distal radius fractures and incidence per 10^4^ individuals for each age group, in Reykjavik in 2004, according to the modified AO classification. (Here, age over 18 is used for comparison with the table from Brogren et al. (2007))

	19–49 years		50–79 years		> 80 years		
	n	Incidence	n	Incidence	n	Incidence	p-value **^a^**
AO type		(95% CI)		(95% CI)		(95% CI)	
Women							
A	19	7 (4–10)	69	49 (37–60)	30	131 (85–178)	< 0.001
B	1	0.4 (0–1.0)	5	3.5 (0.4–7)	1	4 (0–13)	0.2
C	9	3 (1.2–6)	14	10 (5–15)	9	39 (14–65)	< 0.001
Men							
A	14	5 (3–8)	15	12 (6–18)	4	28 (0.6–55)	0.001
B	7	3 (0.7–5)	4	3 (0.1–6)	1	7 (0–21)	0.3
C	10	4 (1.4–6)	9	7.1 (2.4–12)	2	14 (0–33)	0.04

**^a^** Comparing incidence rates among the three age groups using the one-way chi-square test.

### Treatment

The most common initial treatment was casting only (126 patients, 55%), while 91 patients (40%) were treated with closed reduction and casting, and 11 patients (5%) were treated with primary surgery. 6 were treated with external fixation and 5 with open reduction and internal fixation (ORIF). Of the 91 patients primarily treated with closed reduction and casting only, 10 (11%) were treated with early secondary surgery (within 2–3 weeks), involving closed re-reduction and external fixation.

## Discussion

We found that the incidence of distal radius fractures in women aged 50–70 years was considerably lower in 2004 than in 1985 (Robertsson et al. 1990). This difference is factual, since both Icelandic studies covered essentially all fractures in an entire geographic population and were not derived from a chosen sample. Recent studies from Scandinavia (Brogren et al. 2007, Lofthus et al. 2008) have shown the same trend, i.e. a reduced incidence in younger postmenopausal women over the last two decades. The reason for this is unknown, but it is tempting to speculate that it may be related to the widespread use of estrogen hormone replacement therapy (HRT) during the last 15 years of the twentieth century. The authors of a recent study in Oslo estimated that roughly half of the observed decline in incidence in younger postmenopausal women between the 1970s and the 1990s may be attributable to HRT (Mayer et al. 2009). Several studies carried out by the Icelandic Cancer Society between 1986 and 2005 showed that less than 10% of the screened population used HRT prior to 1987 (Eliasson et al. 1998, Armannsdottir et al. 2004). By 2000, this percentage had increased to almost 60% of women aged 50–60 years. Moreover, the average length of HRT treatment also increased during the same period (Armannsdottir et al. 2004). Since the beginning of the twenty-first century, long-term HRT use has decreased substantially, however, and was less than 40% in women aged 50–60 years in 2004–5 (unpublished figures from the Icelandic Cancer Registry), probably due to the publication of new evidence suggesting a higher cardiovascular risk profile associated with HRT. If HRT is associated with reduced fracture incidence, we may see another increase in distal radius fractures in postmenopausal women in the next decade. However, this will probably be difficult to evaluate due to several confounding factors, such as changing lifestyles with exercise, calcium and vitamin D supplementation, and weight control, as well as increased use of corticosteroids and bisphosphonates.

The increase in age-specific fracture incidence in men over 60 between 1985 and 2004 is not statistically significant, but is nonetheless interesting. Although rarely studied, there is some evidence to suggest that male osteoporosis is a growing problem. A recent southern Swedish study found that 60–80-year-old men with distal radius fractures were five times more likely to have osteoporosis based on calcaneal BMD than their non-fractured control counterparts (Atroshi et al. 2009).

The annual incidence of distal radius fractures in Reykjavik was similar in 1985 and 2004 (26 and 27 per 10^4^ individuals) which is consistent with other authors' observations that the rise in incidence between the 1950s and the 1980s has leveled off or that the incidence has decreased in the last two decades (Brogren et al. 2007, Lofthus et al. 2008). The same applies to the total incidence in women in Reykjavik over the 20-year period. However, there appears to be a trend of increase in the total incidence in men in Reykjavik (14 per 10^4^ individuals in 1985 as opposed to 17 per 10^4^ individuals in 2004), and the likeliest explanation is the increased longevity of men—demonstrated by the considerably higher mean age in 2004 than in 1985 (50 vs. 42 years).

The annual incidence of distal radius fractures in women in our study was considerably lower than in Oslo in 1998/99, where the highest incidence in the world was recorded (56 per 104 individuals) (Lofthus et al. 2008). Men, however, had similar annual incidence in both studies, ranking them among the highest in the world. The incidence in women in our study was similar to those in recent studies from Sweden (Brogren et al. 2007) and the United Kingdom (Thompson et al. 2004), but in the latter 2 studies a lower incidence in men was recorded compared to our study.

In the oldest age groups in both sexes, our study supports the findings of other recent studies showing a continuous rise in the incidence of distal radius fractures with age, up to more than 80 years (Mallmin and Ljunghall 1992, Thompson et al. 2004, Brogren et al. 2007, Lofthus et al. 2008). This contrasts with older findings of a plateau, or even a decrease in incidence in the elderly (Owen et al. 1982, Falch 1983, Solgaard and Petersen 1985, Schmalholz 1988, Robertsson et al. 1990). There are several theories regarding this contradiction. The most probable reason is the increasing life expectancy in the developed world, and therefore a relative increase in the number of individuals in the upper part of each 10-year age group as well as an increase in the number of people over the age of 90.

Our study shows less seasonal variation than the earlier Icelandic study, where almost 50% of all fractures occurred during the cold winter months (Robertsson et al. 1990). One possible reason might be milder winters with less icy conditions in Reykjavik during the past decade (the Icelandic Meteorological Office).

Regarding AO classification, we did not find any significant differences in incidence according to age and sex when we compared our results to those of a recent Swedish study (Brogren et al. 2007). However, we found that 32% of fractures in patients over 18 involved the articular surface of the distal radius (AO types B and C), which is higher than the findings of Brogren et al. (2007) (22%). Interestingly, although men accounted for only one third of the total number of fractures in our study, they sustained almost half of the more serious fractures (types B and C). Moreover, a large proportion of all intraarticular fractures occurred in men of working age. In comparison with Swedish men aged 19–79 years who accounted for 29% of all B and C fractures (Brogren et al. 2007), their Icelandic counterparts accounted for 42% of all B and C fractures in our study, which may reflect greater risk-taking behavior among young Icelandic men. The distribution of fractures in women in our study was similar to that reported by Brogren et al. (2007) for Swedish women, with type A fractures predominating (75% and 81%, respectively).

While treatment methods for distal radius fractures have been controversial for decades, in recent years surgical intervention has become more popular in many countries (Arora et al. 2007, Martineau et al. 2007). However, there has been no evidence-based research to determine indications for surgical treatment (Martineau et al. 2007, Handoll and Madhok 2003). A very low proportion of the distal radius fractures were treated surgically in Iceland in 2004. Our study can therefore serve as a foundation for clinical research on long-term outcomes for nonoperatively treated distal radius fractures. Hopefully, it will also contribute to our understanding of the relationship between osteoporosis and estrogen hormone replacement therapy in post-menopausal women.
